# *Gal4* drivers of the geosmin receptor *Or56a* exhibit ectopic expression in the labral sense organ of *Drosophila*

**DOI:** 10.1038/s41598-025-16514-3

**Published:** 2025-08-27

**Authors:** Zepeng Yao

**Affiliations:** Department of Biology, Florida Chemical Senses Institute, McKnight Brain Institute, and Genetics Institute, University of Florida, Gainesville, FL 32611 USA

**Keywords:** *Drosophila*, Geosmin, Or56a, Labral sense organ (LSO), Feeding, Olfactory, Gustatory, Genetics, Neuroscience

## Abstract

The fruit fly, *Drosophila melanogaster*, is a valuable model for studying the mechanisms of chemosensation. The odorant receptor Or56a has been shown to be narrowly tuned to geosmin—a chemical that flies use as a proxy for toxic molds and bacteria—and its activation drives olfactory avoidance behavior. Here, I find that existing *Gal4* drivers using cloned promoter fragments of the *Or56a* gene drive unexpected expression in the labral sense organ (LSO), an internal taste sensory organ within the fly pharynx, in addition to their reported expression in the olfactory antennae. However, the presence of geosmin in sucrose solution does not elicit taste aversion or reduce consumption. Furthermore, a knock-in *Or56a-T2A-Gal4* line newly generated in this study does not drive expression in the LSO. These results suggest that the LSO expression likely reflects ectopic expression from the existing *Or56a-Gal4* drivers rather than the endogenous *Or56a* expression pattern. This study adds to the growing evidence that genetic drivers constructed using cloned promoters may not always faithfully recapitulate endogenous gene expression patterns, which should be taken into consideration when interpreting experimental results.

**Supplementary Information:**

The online version contains supplementary material available at 10.1038/s41598-025-16514-3.

## Introduction

Smell (olfaction) and taste (gustation) are two primary chemical senses that allow animals to detect and interact with chemicals in their environment. Olfaction mainly detects volatile chemicals and helps animals locate foods and mates, as well as recognize predators and other environmental threats. Gustation, on the other hand, is primarily used to evaluate the chemical content of potential food sources, helping animals identify suitable foods and avoid toxic or harmful substances. The olfactory and gustatory systems use distinct molecular receptors, neural circuits, and coding strategies to process chemical information^1^. While the two sensory systems can function independently, their interactions play a significant role in shaping perception and feeding decisions. For example, in humans, the integration of olfactory and gustatory signals contributes to the complex perception of food flavor, which in turn influences dietary choices^2^.

The fruit fly, *Drosophila melanogaster*, has long been used as a model organism to study the olfactory and gustatory systems. While flies and mammals share many similarities in the neural circuits and coding strategies of their olfactory and gustatory systems^3,4^, interestingly, flies use distinct families of proteins as their chemosensory receptors. A family of approximately 60 odorant receptors (ORs) is primarily expressed in olfactory sensory organs—the antennae and maxillary palps—and functions as olfactory receptors^5^. A family of 68 gustatory receptors (GRs) encoded by 60 *Gr* genes is expressed in various taste sensory organs—including the proboscis, legs, and wings—and functions primarily as taste receptors^5^. Lastly, members of a family of approximately 60 ionotropic receptors (IRs) are expressed in olfactory and/or taste organs and function as olfactory or taste receptors^5,6^. The expression patterns of most ORs, GRs, and IRs in chemosensory organs have been well characterized, either by RNA in situ hybridization e.g.,^7–9^ or, more commonly, by using *Gal4* drivers for the respective receptors e.g.,^7,8,10–19^. In the latter approach, the putative promoter sequence of a receptor is cloned and placed upstream of the DNA sequence encoding Gal4, a transcription factor from yeast^20,21^. This construct is then inserted into the fly genome, allowing the cloned promoter to drive *Gal4* expression. When combined with a reporter gene (e.g., green fluorescent protein (GFP)) placed downstream of a *UAS* (upstream activating sequence), the Gal4 protein binds to the *UAS* and activates transcription of the reporter^20,21^. Consequently, the reporter expression reflects the tissue-specific expression pattern driven by the cloned promoter. This approach has been used extensively to map the expression patterns of chemosensory receptors in flies, and in many cases, faithfully recapitulates the endogenous receptor expression pattern as observed through RNA in situ hybridization or reporters and drivers inserted into the receptor gene’s endogenous locus^7–9,16^. However, some *Gal4* drivers have been reported to exhibit ectopic expression, which may result from the presence of regulatory sequences near the insertion site, additional regulatory elements within the cloned promoter, or other unidentified factors^22–24^.

In this study, I found that existing *Gal4* drivers for the odorant receptor Or56a exhibit unexpected expression in the labral sense organ (LSO), an internal taste sensory organ within the *Drosophila* pharynx, in addition to their reported expression in the olfactory antennae. In contrast, a knock-in *Or56a-T2A-Gal4* driver newly generated in this study, in which *Gal4* is inserted into the endogenous *Or56a* locus, does not drive expression in the LSO. These results suggest that the LSO expression displayed by existing *Or56a*-*Gal4* drivers using cloned promoter fragments likely reflects ectopic expression. Consistent with this, the presence of geosmin, a highly specific ligand for Or56a that elicits olfactory avoidance behavior^25^, does not have any measurable effects on food ingestion, supporting the idea that Or56a does not mediate taste aversion in the LSO. Together, my study shows that existing *Or56a-Gal4* drivers constructed using cloned promoters exhibit ectopic expression in the LSO of the pharynx, highlighting the need for caution when interpreting results from behavioral studies that use these drivers to manipulate neural activity. The knock-in *Or56a-T2A-Gal4* generated in this study provides more specific genetic access to the Or56a-expressing olfactory receptor neurons for future research.

## Results

### *Gal4* drivers of the geosmin receptor *Or56a* drive expression in the LSO

**Fig. 1:**
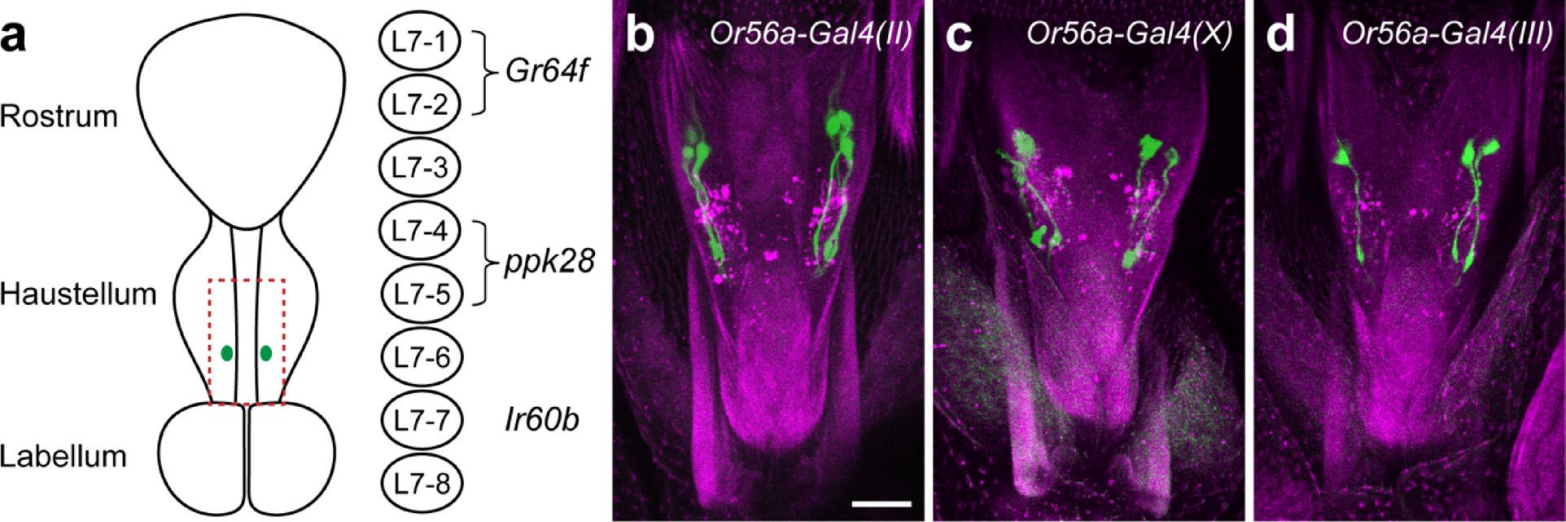
Existing *Or56a-Gal4* drivers exhibit expression in the labral sense organ (LSO). (**a**) A schematic of the *Drosophila* proboscis. The green dots indicate LSO sensilla 7, which contains eight chemosensory neurons (L7-1–8) on each side of the pharynx. L7-1–2 express *Gr64f*, L7-4–5 express *ppk28*, and L7-7 expresses *Ir60b* (see text for details). The red dotted rectangle indicates the approximate area shown in panels (**b**–**d**). (**b**–**d**) All three existing *Or56a-Gal4s* exhibit expression in the LSO. Green color represents GFP expression driven by the indicated *Gal4* drivers, and magenta color represents cuticular autofluorescence. Scale bar = 20 µm.

Geosmin (trans-1,10-dimethyl-trans-9-decalol) is volatile compound produced by toxic molds and bacteria that fruit flies may encounter in their natural feeding environment. While geosmin itself is not toxic to flies, flies use it to detect the presence of toxic molds and bacteria, and have evolved a highly specific odorant receptor, Or56a, to detect it^25^. Activation of the Or56a-expressing olfactory receptor neurons (ORNs) and their downstream circuits by geosmin deters the flies from feeding or laying eggs on the substrate^25^. *Gal4* drivers have been previously generated by two independent groups to genetically target the Or56a-expressing ORNs, which are housed in the fly antennae^10,11^. While examining the expression pattern of these *Or56a-Gal4* drivers, I noticed that, in addition to the expected expression in antennae, they also drive expression in the labral sense organ (LSO) in the pharynx (Fig. 1). The LSO is an internal sensory organ in the pharynx located in the haustellum of the fly proboscis (Fig. 1a), containing nine pairs of sensilla^17,26,27^. Based on their morphology, *Or56a-Gal4s* drive expression in sensillum 7, which contains eight chemosensory neurons on each side of the pharynx^17,26,27^ (Fig. 1a). Of the three publicly available *Or56a-Gal4* drivers^10,11^, *Or56a-Gal4(II)* labels three pairs of LSO neurons (Fig. 1b), *Or56a-Gal4(X)* labels two pairs of LSO neurons (Fig. 1c), while the expression of *Or56a-Gal4(III)* is more variable, labeling one to two pairs of LSO neurons (Fig. 1d). Therefore, all three existing *Or56a-Gal4* drivers, generated by two independent groups^10,11^, drive unexpected expression in LSO neurons in the fly pharynx.

### *Or56-Gal4(II)* drives expression in a group of poorly characterized LSO neurons

**Fig. 2:**
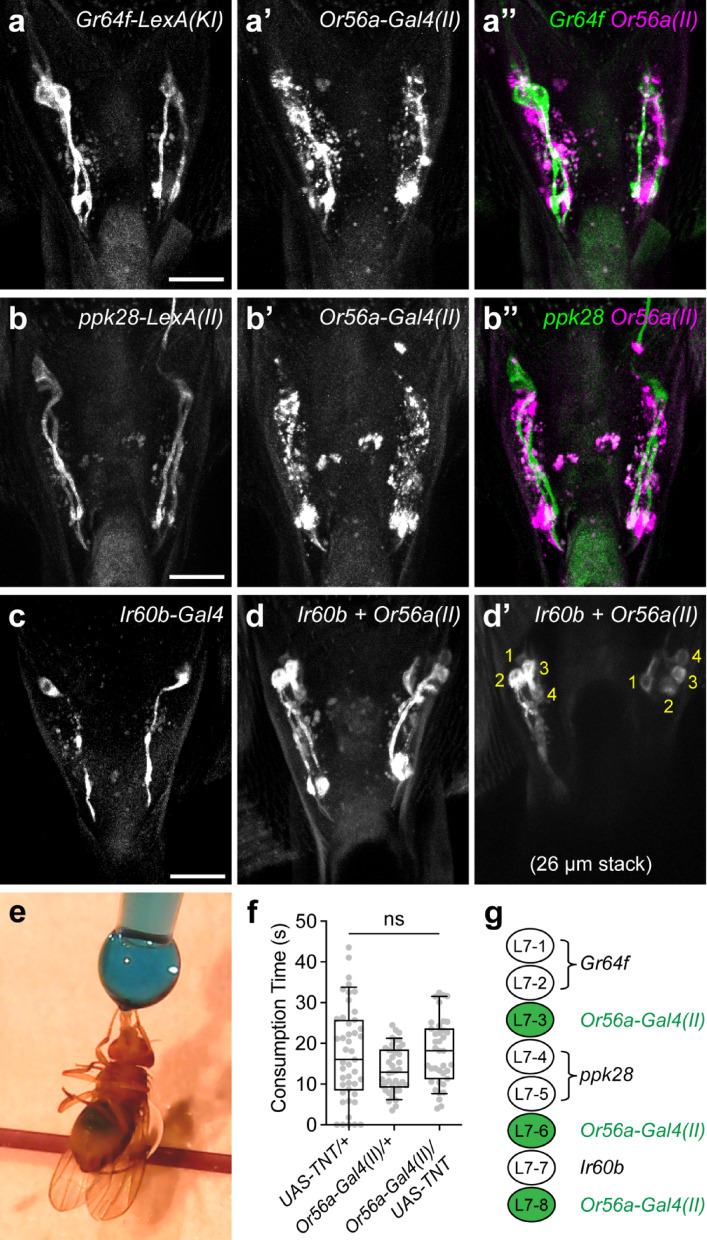
*Or56-Gal4(II)* labels a group of poorly characterized LSO neurons. (**a**–**a’’**) *Gr64f-LexA(KI)* and *Or56a-Gal4(II)* label distinct populations of LSO neurons. *Gr64f-LexA(KI)* drove myr-GFP expression (**a** and **a’’** green), while *Or56a-Gal4(II)* drove CD8-tdTomato expression (**a’** and **a’’** magenta). Scale bar = 20 µm. (**b**–**b’’**) *ppk28-LexA(II)* and *Or56a-Gal4(II)* label distinct populations of LSO neurons. *ppk28-LexA(II)* drove myr-GFP expression (**b** and **b’’** green), while *Or56a-Gal4(II)* drove CD8-tdTomato expression (**b’** and **b’’** magenta). Scale bar = 20 µm. (**c**) mCD8-GFP expression driven by *Ir60b-Gal4* labels one pair of LSO neurons. Scale bar = 20 µm. (**d**–**d’**) mCD8-GFP expression driven by both *Ir60b-Gal4* and *Or56a-Gal4(II)* labels four pairs of LSO neurons. (**d**) shows the full confocal projection (64 µm), while (**d’**) shows a 26 µm stack to better visualize the cell bodies (labeled 1–4 on each side). (**e**) Measuring sucrose consumption time for individual flies using the temporal consumption assay. (**f**) Consumption time of control flies and flies with *Or56a(II)* LSO neurons silenced using tetanus toxin (TNT). All flies were food-deprived for approximately 24 h and tested with 300 mM sucrose. For box plots: whiskers = 10th–90th percentile, box = 25th–75th percentile, and line within box = median. Dots represent individual data points. N = 38–45 flies/genotype; one-way ANOVA followed by Tukey’s multiple comparison tests, ns = not significant. (**g**) Summary of the *Or56a-Gal4(II)* expression pattern in the LSO sensillum 7.

Given that *Or56a-Gal4(II)* labels the most LSO neurons among the three *Or56a-Gal4* drivers (Fig. 1b–d), I decided to focus on this driver and further characterize the LSO neurons that it labels. The LSO sensillum 7 (L7) contains eight pairs of chemosensory neurons, named L7-1 to L7-8 (Fig. 1a)^17,26,27^. Recent studies have revealed that multiple gustatory receptors (GRs) and ionotropic receptors (IRs) are expressed in these L7 neurons. For example, *Gr64f* and *Gr43a* are expressed in L7-1 and L7-2 (Fig. 1a), which play a role in regulating the ingestion of sugars and amino acids^28,29^. The water receptor *ppk28*^30,31^ is expressed by L7-4 and L7-5^17^, suggesting that they may regulate water ingestion. The ionotropic receptor *Ir60b* is expressed by a single pair of L7 neurons, L7-7, which suppresses the ingestion of high salt and sucrose^17,29,32,33^. To address which L7 neurons *Or56a-Gal4(II)* labels, double-labeling experiments were performed. CD8-tdTomato was expressed in the *Or56a(II)* LSO neurons using *Or56a-Gal4(II)*, and myr-GFP was independently expressed in L7-1 and L7-2 neurons using a knock-in *Gr64f-LexA(KI)*^34^ (Fig. 2a–a’’). Expression of CD8-tdTomato resulted in punctate structures in the *Or56a(II)* LSO neurons, making it difficult to clearly observe their morphology (Fig. 2a’). However, there was clearly no detectable CD8-tdTomato expression in the *Gr64f* L7-1 and L7-2 neurons (Fig. 2a’’), indicating that the *Or56a(II)* LSO neurons are distinct from the L7-1 and L7-2 neurons that express *Gr64f*. Similarly, I performed double-labeling experiments with *Or56a-Gal4(II)* and *ppk28-LexA(II)* and found that the *Or56a(II)* LSO neurons are also distinct from the L7-4 and L7-5 neurons that express the water receptor *ppk28* (Fig. 2b–b’’). Lastly, I investigated whether the *Or56a(II)* LSO neurons include the *Ir60b-*expressing L7-7 neurons. Because of the lack of an *Ir60b-LexA* driver at the time of the experiment, double-*Gal4* labeling experiments were performed. *Ir60b-Gal4* labels a single pair of L7 neurons (Fig. 2c)^17,29,32,33^, while *Or56a-Gal4(II)* labels three pairs of LSO neurons (Fig. 1b). I combined both *Gal4s* to drive mCD8-GFP expression and carefully counted the number of LSO neurons labeled (Fig. 2d–d’). A total of four pairs of LSO neurons were labeled (Fig. 2d’), indicating that the *Or56a(II)* LSO neurons are also distinct from the *Ir60b-*expressing L7-7 neurons. Additionally, it was reported that blocking synaptic transmission of the *Ir60b* L7-7 neurons by expressing tetanus toxin (TNT) resulted in increased sucrose consumption^32^. Using a largely identical consumption assay (Fig. 2e), I found that TNT expression in the *Or56a(II)* LSO neurons had no effects on sucrose consumption (Fig. 2f). These results further suggest that the *Or56a(II)* LSO neurons are distinct from the *Ir60b-*expressing L7-7 neurons. Taken together, the *Or56a-Gal4(II)* does not label the *Gr64f*-expressing L7-1 and L7-2 neurons, the *ppk28*-expressing L7-4 and L7-5 neurons, or the *Ir60b*-expressing L7-7 neurons (Fig. 2g). Therefore, it labels the L7-3, L7-6, and L7-8 neurons (Fig. 2g) and provides genetic access to these relatively less well characterized LSO neurons.

### The presence of geosmin in sucrose food does not affect consumption

**Fig. 3:**
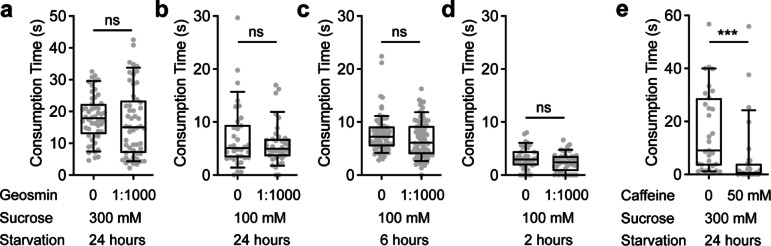
The presence of geosmin has no measurable effects on sucrose consumption. (**a**–**e**) Consumption time of immobilized *Canton-S* flies measured by the temporal consumption assay. In (**a**–**d**), flies were food-deprived for the indicated durations and tested with the indicated concentrations of sucrose solution, either without or with 1:1000 geosmin. In (**e**), flies were food-deprived for 24 h and tested with 300 mM sucrose, either without or with 50 mM caffeine. For box plots: whiskers = 10th–90th percentile, box = 25th–75th percentile, and line within box = median. Dots represent individual data points. N = 31–77 flies/genotype; Mann–Whitney test, ns = not significant; ***p < 0.001.

LSO neurons play important roles in regulating the ingestion of various compounds, including sugars, amino acids, salts, and bitters^28,29,32,33,35,36^. My findings that drivers of the geosmin receptor Or56a are expressed in LSO neurons led me to investigate if the *Or56a(II)* LSO neurons (L7-3, L7-6, and L7-8) can detect geosmin and suppress the ingestion of food containing geosmin, a compound used by flies as a proxy for toxic microbes^25^. Using a capillary feeder (CAFE) assay^37^, where free-moving flies fed from food-containing capillaries, a previous study showed that the presence of geosmin (0.1%) in sucrose solution deterred flies from feeding from it^25^. Given that geosmin is an aversive odorant^25^, the observed feeding suppression was likely due to flies avoiding the geosmin-containing capillary. However, it is also possible that geosmin elicits an aversive taste, detected by the *Or56a(II)* LSO neurons, which (further) inhibits ingestion. To better investigate this possibility, I measured food ingestion using immobilized flies (Fig. 2e) (see Methods for details), which should largely eliminate the effects of geosmin on olfaction-guided foraging behavior. Interestingly, the presence of 0.1% geosmin in 300 mM sucrose solution had no measurable effects on consumption by flies fasted for 24 h (Fig. 3a). I reasoned that 300 mM sucrose might be quite appetitive to the flies, and that flies fasted for 24 h might be very motivated to feed, both of which might mask the effects of geosmin. Therefore, I repeated the experiments with a lower concentration of sucrose (100 mM) and flies fasted for shorter periods of time (24, 6, and 2 h) (Fig. 3b–d). These conditions indeed seemed to decrease sucrose ingestion time (compare Fig. 3b–d to Fig. 3a). However, in every condition tested, the presence of geosmin had no measurable effects on the consumption of sucrose solution (Fig. 3a–d). In contrast, the addition of 50 mM caffeine, a bitter compound, significantly suppressed sucrose consumption (Fig. 3e). Taken together, these results suggest that even at a high concentration, geosmin is unlikely to elicit an aversive taste for flies.

### A knock-in *Or56a-T2A-Gal4* driver does not drive expression in LSO neurons

**Fig. 4:**
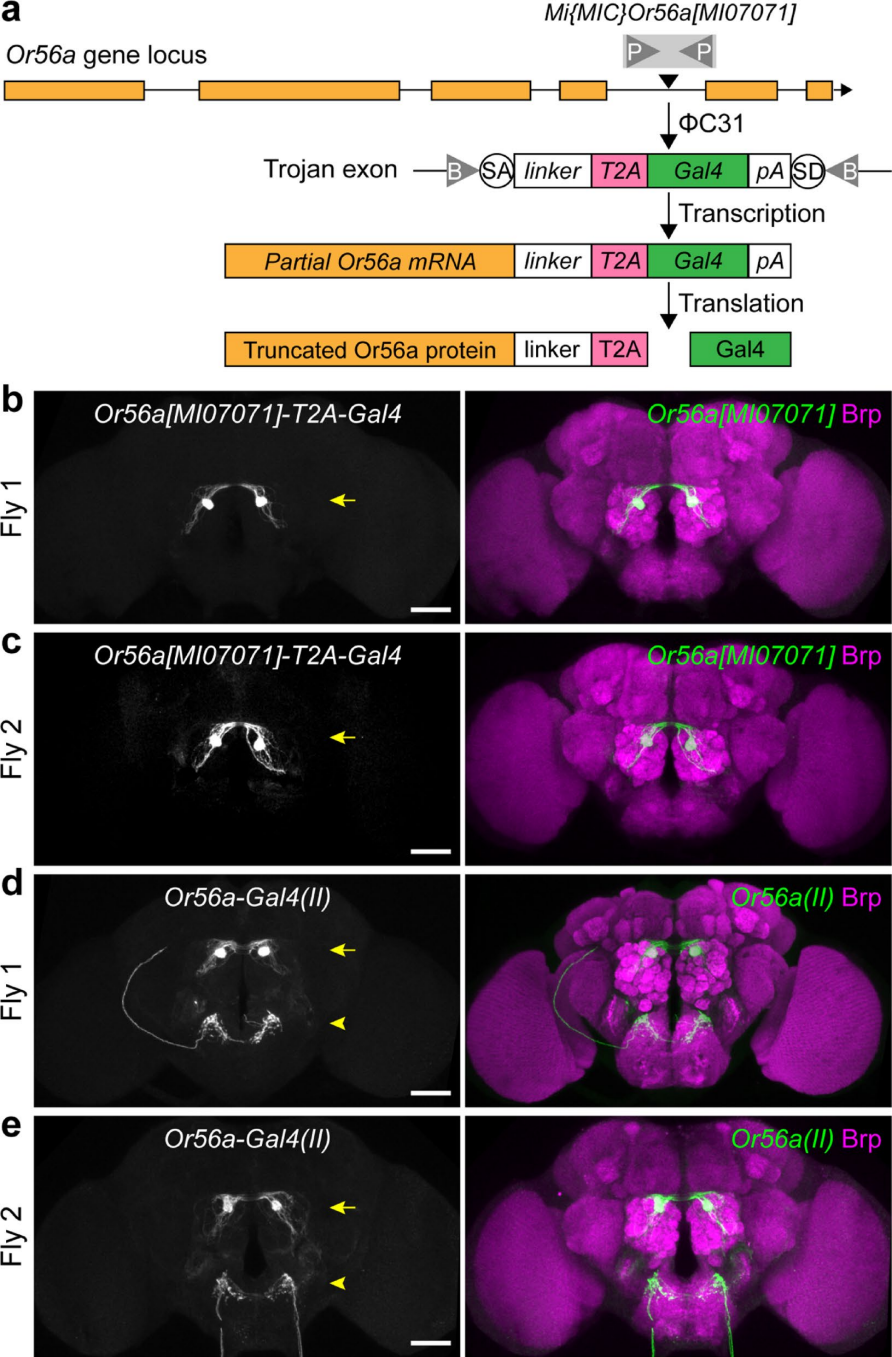
A knock-in *Or56a-T2A-Gal4* driver does not drive expression in LSO neurons. (**a**) Schematic illustrating the generation of *Or56a[MI07071]-T2A-Gal4* using the Trojan Exon approach (see text for details). P: attP site; B: attB site; SA: splice acceptor site; SD: splice donor site; pA, polyadenylation signal. (**b**, **c**) Immunostaining of mCD8-GFP expression driven by *Or56a[MI07071]-T2A-Gal4* in two representative brains. Arrows indicate expression in the antennal lobes. (**d**, **e**) Immunostaining of mCD8-GFP expression driven by *Or56a-Gal4(II)* in two representative brains. Arrows indicate expression in the antennal lobes; arrowheads indicate expression in the dorsal subesophageal zone (SEZ), where LSO neurons project. In (**b**–**e**), immunostaining of mCD8-GFP expression is shown in black and white in the left panels and in green in the right panels. Immunostaining for Bruchpilot (Brp), which labels brain neuropils, is shown in magenta. Scale bars = 50 µm.

My above findings that the presence of geosmin does not affect the ingestion of sucrose food led me to reconsider whether the endogenous Or56a protein is expressed in the LSO. The *Or56a-Gal4* drivers tested above (Fig. 1) were generated by placing the putative promoter region of the *Or56a* gene upstream of the *Gal4* sequence and inserting the construct randomly into the fly genome^10,11^. This may not faithfully recapitulate the endogenous expression pattern of the *Or56a* gene. To more accurately report the endogenous expression pattern of *Or56a*, the Trojan Exon approach was used^38^. In brief, a *MiMIC* cassette^39^ in the coding intronic region of *Or56a* was replaced with a Trojan exon containing the *T2A-Gal4* sequence (see Methods for details) (Fig. 4a). This resulted in the translation of Gal4 protein only in cells that normally express the endogenous Or56a protein (Fig. 4a). This knock-in *Or56a-T2A-Gal4* is expected to faithfully report the endogenous expression pattern of the *Or56a* gene.

I used the knock-in *Or56a-T2A-Gal4* to drive mCD8-GFP expression in *Or56a*-expressing cells and found that there was no mCD8-GFP expression in the LSO. In contrast, there was mCD8-GFP expression in the antenna, where the Or56a olfactory receptor neurons (ORNs) are located. Indeed, when brain expression was examined using immunostaining, mCD8-GFP was exclusively expressed in a single pair of glomeruli in the antennal lobes (Fig. 4b,c, arrows)—the DA2 glomeruli—to which the Or56a ORNs send their axonal projections^10,11,25^. In contrast, in brains where mCD8-GFP expression was driven by *Or56a-Gal4(II)*, expression was observed not only in the DA2 glomeruli (Fig. 4d,e, arrows), but also in the dorsal subesophageal zone (SEZ) (Fig. 4d,e, arrowheads), where LSO neurons project^17^. These results strongly suggest that the endogenous Or56a is only expressed in the Or56a ORNs and not in LSO neurons, which is consistent with my above findings that geosmin is unlikely to be detected by LSO neurons to elicit an aversive taste.

## Discussion

In this study, I found that all three publicly available *Gal4* drivers of the geosmin receptor *Or56a* are expressed in the LSO sensillum 7 (Fig. 1). This is unexpected, given that Or56a is an odorant receptor known to function in the antennae to detect geosmin vapor^25^, whereas the LSO sensillum 7 is a taste sensillum in the pharynx that detects chemicals in the passing food during ingestion^17,26,27^. However, it was reported that mammalian taste cells express functional olfactory receptors^40^, and *Drosophila* sweet- and bitter-sensing gustatory receptor neurons (GRNs) can directly respond to odors^41^, suggesting that it is possible for taste neurons to express functional olfactory receptors to detect odors. Given that Or56a is narrowly tuned to geosmin—detecting geosmin with high specificity and affinity—and that geosmin is used by flies to indicate the presence of toxic microbes^25^, I reasoned if the LSO indeed expresses a functional Or56a receptor, the activation of LSO Or56a by geosmin in the food would warn the flies of potential toxic microbes and suppress food ingestion. However, the presence of geosmin, even at a high concentration, in sucrose solutions has no measurable effects on sucrose consumption (Fig. 3), raising the possibility that the endogenous Or56a protein is not expressed or not functional in the LSO. Indeed, when I generated a knock-in *Or56a-T2A-Gal4* using the Trojan Exon approach^38^, I found that it did not drive expression in the LSO, providing strong evidence that *Or56a* is not endogenously expressed by LSO neurons (Fig. 4). These results suggest that the reported effects of geosmin on suppressing feeding in a capillary feeder (CAFE) assay should be largely due to its aversive smell, causing flies to avoid the geosmin-containing food, as previously suggested^25^. Once the foraging component is eliminated (as by immobilizing flies and manually presenting food to them in this study), the presence of geosmin and its aversive smell appears to have little influence on the fly’s feeding.

It is unclear why all three *Or56a-Gal4s* drive ectopic expression in the LSO. *Or56a-Gal4(X)* and *Or56a-Gal4s(II)* use a 5.385 kb promoter fragment upstream of the *Or56a* gene to drive *Gal4* expression^10^, while *Or56a-Gal4s(III)* uses a 5.286 kb promoter fragment^11^. Since these *Or56a-Gal4* constructs were randomly inserted into different locations in the genome (on chromosomes X, II, and III, respectively) but all drive expression in the LSO, it is unlikely that regulatory sequences near the insertion sites are responsible for the ectopic LSO expression. Instead, it is more likely that some regulatory sequence(s) within the cloned promoter fragments cause this ectopic expression. The cloned promoter fragments do not contain any open reading frames—the closest gene upstream of *Or56a*, *odorant-binding protein 56 g* (*Obp56a*), is approximately 12 kb away. It might be of interest for future studies to identify the specific regulatory sequence(s) responsible for the ectopic expression in the LSO. Nevertheless, these *Or56a-Gal4s*—particularly *Or56a-Gal4(II)*—provide genetic access to a group of relatively less well characterized LSO neurons (L7-3, L7-6, and L7-8) (Fig. 2). These LSO neurons express a few gustatory receptors (including Gr2a, Gr23a, Gr57a, and Gr93d) and ionotropic receptors (including Ir56a, Ir67c, Ir100a, and co-receptors Ir25a and Ir76b)^17^. However, many of these receptors are also expressed in other sensory organs, such as the legs, labella, and other parts of the pharynx, limiting their use as specific genetic drivers for the LSO neurons. In contrast, combining *Or56a-Gal4(II)* and *Orco-Gal80*^42^—the latter expected to suppress Gal4 activity in *Or56a* ORNs—should provide highly specific genetic access to the L7-3, L7-6, and L7-8 neurons and may facilitate future studies on their functions.

The *Or56a-T2A-Gal4* generated in this study will be useful for future research. First, it is a knock-in *Gal4* driver faithfully reflecting the endogenous *Or56a* expression pattern. It provides highly specific genetic access to the *Or56a* ORNs without causing ectopic LSO expression displayed by the currently available *Or56a-Gal4s*^10,11^. Second, the *Or56a-T2A-Gal4* generated here using the Trojan Exon approach^38^ results in translation of truncated Or56a proteins (Fig. 4a) and therefore is expected to cause a partial or total loss-of-function of *Or56a*. This is different from strategies that insert *T2A-Gal4* at or near the 3’ end of a gene, which can leave the endogenous gene functionally intact while expressing *Gal4* in the gene’s endogenous pattern^43^. While further characterization is required, the *Or56a-T2A-Gal4* generated here represents an additional mutant allele of the *Or56a* gene, in addition to the *Or56a* knockout allele^44^, for future functional studies. Lastly, I note the potential use of *Or56a-T2A-Gal4* to express exogenous olfactory receptors—including those from *Drosophila melanogaster*, other *Drosophila* species, or even other insects such as mosquitoes and ants—in the *Or56a* ORNs to characterize their odorant ligands. Given that Or56a is narrowly tuned to geosmin^25^, as long as geosmin is not a ligand for the tested olfactory receptor, the presence of full-length Or56a proteins should not confound the results. Similar strategies have been employed in previous studies using *Or22a*^*Gal4*^ and *Or67d*^*Gal*^^4,45,46^. Given that activation of *Or56a* ORNs elicits robust avoidance behavior^25^, it might be possible to screen for odorant ligands of the tested olfactory receptor using behavioral assays, in addition to electrophysiological recordings.

## Methods

### Fly strains and genetics

Fruit flies (*Drosophila melanogaster*) were reared on standard cornmeal-yeast-molasses media at 25 °C under a 12-h light:12-h dark cycle. The genotypes of the flies used in the figures are listed in Supplementary Table S1. The following fly strains were used, most of which are available from the Bloomington *Drosophila* Stock Center (BDSC). *Canton-S* (maintained in Kristin Scott’s lab), *Or56a-Gal4(X)* (RRID:BDSC_9987), *Or56a-Gal4(II)* (RRID:BDSC_9988), *Or56a-Gal4(III)* (RRID:BDSC_23896), *UAS-EGFP(5a.2)* (RRID:BDSC_5431), *UAS-mCD8::GFP.L(LL4)* (RRID:BDSC_5136), *UAS-mCD8::GFP.L(LL5)* (RRID:BDSC_5137), *UAS-CD8-tdTomato*^47^, *13XLexAop2-IVS-myr::GFP(su(Hw)attP1)* (RRID:BDSC_32212), *Gr64f-LexA(knock-in)* (RRID:BDSC_93445), *ppk28-LexA(II)*^47^, *UAS-mCD8::GFP.L(LL5); Ir60b-Gal4.K(attP2)* (RRID:BDSC_60710), and *UAS-TNT* (RRID:BDSC_28838).

The knock-in *Or56a-T2A-Gal4* driver was generated using the Trojan Exon approach, as described in^38^. In brief, the *Mi{MIC}Or56a[MI07071]* strain (RRID:BDSC_42202), which contains a *MiMIC* cassette^39^ in the coding intronic region of *Or56a*, was sequentially crossed to *lox(Trojan-GAL4)* × *3(11)* (RRID:BDSC_60311) and *hs-Cre,vas-dΦC31* (RRID:BDSC_60299) to induce the replacement of the *MiMIC* cassette with the Trojan exon containing the *T2A-Gal4* sequence. Single male offspring were crossed to *UAS-2xEYFP; Sp-1/CyO; Dr[1]/TM3,Sb* (RRID:BDSC_60291) and screened for EYFP expression in the antennae and/or in the antennal lobes. One *Or56a-T2A-Gal4* line was recovered and balanced.

### Confocal imaging of the proboscis

The analysis of reporter expression in the labral sense organ (LSO) of the proboscis was done similar to what was described in^32^. In brief, female flies expressing GFP or other fluorescent proteins were decapitated, and their heads and proboscises were mounted on a microscope slide in 50% glycerol. The samples were immediately imaged using a Zeiss LSM 780 confocal microscope equipped with a Zeiss Plan-APOCHROMAT 20x/1.0 water objective. GFP fluorescence was imaged using a 488 nm laser, and tdTomato fluorescence was imaged using a 561 nm laser. For Fig. 1, where flies only had GFP expression, the autofluorescence of the cuticle was imaged using a 561 nm laser, and it was subtracted from the GFP channel to better visualize the GFP-expressing neurons. Image processing and calculations were performed using Fiji/ImageJ (https://imagej.net/software/fiji/).

### Brain immunohistochemistry and confocal imaging

Immunostaining of whole-mount *Drosophila* brains was performed as previously described^48^ with minor modifications. Fly heads with cuticles gently torn open using forceps were fixed in 4% paraformaldehyde in phosphate buffered saline (PBS) for 1 h at room temperature. After three washes with PBS, the heads were transferred to a sylgard-coated petri dish filled with PBS for dissection. The brains were dissected using fine forceps and transferred to a 2 mL round-bottom tube filled with PBST (PBS with 0.3% Triton X-100). After enough brains were collected, PBST was removed, and the brains were blocked with 5% normal goat serum in PBST (block solution) for 1 h at room temperature, then incubated with primary antibodies in block solution at 4 °C for 2–3 days. After five 15-mininute washes in PBST, the brains were incubated with secondary antibodies in block solution at 4 °C for 1–2 days. After five 15-min washes in PBST, followed by 1–2 exchanges of PBS, the brains were mounted on poly-L-lysine-coated coverslips in PBS and dehydrated in a graded glycerol series (30%, 50%, and 70% glycerol in PBS for 5 min each). The final glycerol solution was replaced with Vectashield Antifade Mounting Medium (H-1000) for imaging and storage. The primary antibodies used were rabbit anti-GFP polyclonal antibody (Invitrogen A-11122, 1:1000 dilution) and mouse anti-Brp (nc82) monoclonal antibody (Developmental Studies Hybridoma Bank nc82-c, 1:500 dilution), and the secondary antibodies used were goat anti-rabbit Alexa 488 (Invitrogen A-11034, 1:1000 dilution) and goat anti-mouse Alexa 568 (Invitrogen A-11031, 1:1000 dilution).

The samples were imaged using a Zeiss LSM 780 confocal microscope equipped with a Zeiss Plan-APOCHROMAT 20x/1.0 water objective. Alexa 488 fluorescence was imaged using a 488 nm laser, and Alexa 568 fluorescence was imaged using a 561 nm laser. Image brightness and contrast were adjusted using Fiji/ImageJ (https://imagej.net/software/fiji/).

### Temporal consumption assay

The temporal consumption assay was performed as previously described^48,49^. Adult mated female flies, 4–14 days old, were food-deprived in a plastic vial containing a piece of wet Kimwipe tissue for the indicated duration. Flies were anesthetized with CO_2_, mounted with their dorsal thorax affixed to a glass microscope slide using nail polish, and allowed to recover in a humidified chamber for approximately 2–3 h. Individual flies were presented with a drop of solution containing the indicated compound(s) (supplemented with 0.25 mg/mL FD&C No. 1 blue dye for visualization) from a 200 µL pipette tip attached to a 1 mL syringe at least 10 times and allowed to ingest the solution until consumption stopped. The total consumption time for each fly was manually recorded using a stopwatch. Geosmin (UC18-10MG) and caffeine (C0750-5G) were purchased from MilliporeSigma.

## Supplementary Information

Below is the link to the electronic supplementary material.

Supplementary Material 1

## Data Availability

Data reported in this paper will be made available upon request to Z.Y.
