# Supplementary material for: Gal4 drivers of the geosmin receptor Or56a exhibit ectopic expression in the labral sense organ of Drosophila
Source: Sci Rep. 2025 Aug 27;15:31651. doi: 10.1038/s41598-025-16514-3 (PMC12391301; doi:10.1038/s41598-025-16514-3)
Supplement: Supplementary file 1 — Supplementary Material 1 [file 41598_2025_16514_MOESM1_ESM.pdf]

Gal4 drivers of the geosmin receptor _Or56a_ exhibit ectopic expression in the labral sense organ of _Drosophila_

Zepeng Yao*

Department of Biology, Florida Chemical Senses Institute, McKnight Brain Institute, and Genetics Institute, University of Florida, Gainesville, FL 32611

* Correspondence: zepengyao@ufl.edu

Table S1: Fly Genotypes Used in the Figures

Figure	Abbreviation	Full Genotype
1b	*Or56-Gal4(II)*	*; Or56a-Gal4.F(63.3)/UAS-EGFP(5a.2); +/TM2*
1c	*Or56-Gal4(X)*	*Or56a-Gal4.F(63.2B)/+; UAS-mCD8::GFP.L(LL5)/+;*
1d	*Or56-Gal4(III)*	*; UAS-mCD8::GFP.L(LL5)/+; Or56a-Gal4.C(113t53.2)/TM2*
2a-a’’	*Gr64f Or56a(II)*	*UAS-CD8-tdTomato/+; Or56a-Gal4.F(63.3)/(Bl or CyO); Gr64f-LexA(knock-in)/13XLexAop2-IVS-myr::GFP(su(Hw)attP1)*
2b-b’’	*ppk28 Or56a(II)*	*UAS-CD8-tdTomato/+; Or56a-Gal4.F(63.3)/ppk28-LexA(II); 13XLexAop2-IVS-myr::GFP(su(Hw)attP1)/(TM2 or TM6B)*
2c	*Ir60b-Gal4*	*; UAS-mCD8::GFP.L(LL5); Ir60b-Gal4.K(attP2)*
2d-d’	*Ir60b + Or56a(II)*	*UAS-mCD8::GFP.L(LL4)/+; Or56a-Gal4.F(63.3)/UAS-mCD8::GFP.L(LL5); Ir60b-Gal4.K(attP2)/+*
2f	*UAS-TNT/+*	*; UAS-TNT/+;*
	Or56-Gal4(II)/+	*; Or56a-Gal4.F(63.3)/+;*
	Or56-Gal4(II)/ UAS-TNT	*; Or56a-Gal4.F(63.3)/UAS-TNT;*
3a-e		*Canton-S*
4b-c	*Or56a[MI07071]*	*; Or56a[MI07071]-T2A-Gal4/UAS-mCD8::GFP.L(LL5);*
4d-e	*Or56a(II)*	*; Or56a-Gal4.F(63.3)/UAS-mCD8::GFP.L(LL5);*
